# Intermittent Claudication in Physiotherapists' Practice

**DOI:** 10.1155/2019/2470801

**Published:** 2019-09-18

**Authors:** Anna Spannbauer, Maciej Chwała, Tomasz Ridan, Arkadiusz Berwecki, Piotr Mika, Anita Kulik, Małgorzata Berwecka, Maria T. Szewczyk

**Affiliations:** ^1^Department of Experimental and Clinical Surgery, Jagiellonian University Medical College, Krakow, Poland; ^2^The Bonifratri Order Hospital of Saint John Grande in Krakow, Krakow, Poland; ^3^Department of Vascular Surgery, University Hospital in Krakow, Krakow, Poland; ^4^Department of Kinesitherapy, University of Physical Education, Krakow, Poland; ^5^Department of Clinical Rehabilitation, University of Physical Education, Krakow, Poland; ^6^Poznan University School of Physical Education, Gorzow Wielkopolski, Poland; ^7^Department of Bone and Joint Diseases, Jagiellonian University Medical College, Krakow, Poland; ^8^Department of Perioperative Nursing, Department of Surgical Nursing and Chronic Wound Care, Nicolaus Copernicus University in Torun, Collegium Medicum in Bydgoszcz, Bydgoszcz, Poland; ^9^Department of Vascular Surgery and Angiology, Nicolaus Copernicus University in Torun, Collegium Medicum in Bydgoszcz, Bydgoszcz, Poland

## Abstract

Intermittent claudication is a symptom of atherosclerosis of the lower limbs (peripheral arterial disease (PAD)) and is characterized by pain and cramps of lower limb muscles during exercise. Claudication leads to a reduction in physical activity of patients. PAD is a systemic disease. Atherosclerotic lesions located in the arteries of the lower limbs not only pose the risk of the ischemic limb loss, but above all, they are an important prognostic factor. Patients with claudication are at significant risk of cardiovascular complications such as infarcts or strokes. Comprehensive rehabilitation of patients with intermittent claudication based on the current TASC II (Inter-Society Consensus for the Management of Peripheral Arterial Disease) guidelines, ESC (European Society of Cardiology) guidelines, and AHA (American Heart Association) guidelines includes supervised treadmill training, training on a bicycle ergometer, Nordic Walking, resistance exercises of lower limb muscles, and exercises of upper limbs. A trained, educated, and motivated patient has a chance to improve life quality as well as life expectancy.

## 1. Introduction

One of the most common causes of disease and death in the Western world is atherosclerosis. The process is characterized by the formation of atherosclerotic plaques that narrow the lumen of the arteries, and its clinical symptoms depend on the location of the lesions. PAD prevalence varies widely between countries, increases sharply with age, and has a relation to ethnicity [1]. Atherosclerosis is considered a systemic disease. It may present as coronary artery disease, cerebrovascular disease, and renal artery stenosis or peripheral arterial disease. The latter disorder (PAD) consists in narrowing and finally total occlusion of the main vessels supplying the lower limbs [2]. This results in pain and disturbances in the economics of walking, which limits the ability to ambulate and decreases physical activity of the patients. Hence, it forms a significant problem for the physiotherapist. The rehabilitation is complex. It must take into account not only the risk of amputation of ischemic lower limbs and reduced quality of life but above all the fact that limb ischemia is an important prognostic factor of general cardiovascular complications [3]. Therefore, kinesitherapy in patients with atherosclerotic lower limb ischemia apart from rehabilitation of locomotive disturbances must take into account the elements of cardiac rehabilitation [4].

### 1.1. Intermittent Claudication

PAD often manifests as intermittent claudication. It is characterized by cramp and pain of a given muscle group. Discomfort occurs only during exercise and intensifies gradually as one continues to walk until it forces complete stop. Effort-related lower limb muscle pain is associated with transient ischemia (IIa and IIb degrees of Fontaine classification). The patient with intermittent claudication does not feel any discomfort at rest because the blood flow and oxygen supply in the limb are then replenished. During exercise, narrowed or occluded arteries supplying the muscles of the lower limbs limit the necessary increase in blood flow, which results in disproportions between metabolic demand and oxygen supply and is associated with the occurrence of claudication [5]. As a result of muscle ischemia, anaerobic metabolic products are released. Anaerobic glycolysis resulting from limited oxygen supply is accompanied by the increase in lactic acid concentration and depletion of adenosine triphosphate (ATP) and phosphocreatine stocks, causing pain [6]. The symptom of claudication, also called the symptom of “shop displays” or “hungry muscle pain,” is so characteristic for PAD that proper diagnosis of the disease can usually be made based solely on properly collected anamnesis [7].

Intermittent claudication limits the locomotive abilities and also makes it difficult for the patient to actively participate in personal, social, and professional life and is one of the causes of disability of middle-aged and elderly people [8]. Fontaine's scale is used for staging of the atherosclerotic disease of lower extremities. Absence of or minimal clinical symptoms in the form of tingling, numbness, and cold sensitivity define stage I. Stage IIa is intermittent claudication with the distance above 200 m, whereas stage IIb is below 200 m. Stage III accounts for rest pain, and grade IV comprises ulcers of gangrene and necrosis. Stages III and IV are referred to as critical limb ischemia [9].

### 1.2. Evaluation of the Patient with PAD by the Physiotherapist

The basic examination of patients with PAD consists of palpation of the pulse in the typical sites on the lower limb, i.e., in the inguinal and popliteal regions and dorsal region of the foot and medial ankle. The lack of a palpable pulse indicates a significant narrowing or occlusion in the arterial system proximally to the examined area [10].

There are two determinants of the patient's ability to ambulate. The maximal claudication distance (MCD) is defined as the distance after which the patient is forced to stop because of severe pain and muscle cramps. Another one, i.e., pain-free walking distance (PFWD), is the distance traveled before any pain occurs. A correctly estimated distance is an important element in the decision of a vascular surgeon or angiologist regarding further medical or surgical treatment. A distance of about 100 m and below prequalifies the patient for invasive treatment [11].

The degree of impairment of blood supply to the limb is assessed by measurement of the ABI (ankle-brachial index). The ABI is expressed as the ratio of the systolic pressure measured in the area of the ankle and the systolic pressure measured in the arm. Under physiological conditions, the ABI in the supine position is close to 0.9–1.4. In patients with PAD, ABI values fall below 0.9, and below 0.4, it correlates with critical limb ischemia [12]. It should however be remembered that, in a subgroup of patients with diabetes or renal insufficiency, false negatives can be expected. It is due to the so-called “stiffness” of small artery walls and their resistance to compression [13, 14]. From the general health's point of view, the reduced ABI also correlates with cardiovascular events. Chronic lower limb ischemia is closely related to myocardial ischemic disease and cerebral artery disease. An ABI less than or equal to 0.9 is associated with a 3- to 6-fold increase in the risk of cardiovascular mortality. This risk correlates with the ABI reduction rate—the lower the ABI, the greater the risk [15].

The latest findings of the European Society for Vascular Medicine in May 2019 contained in the Second European Agreement prove that classic PAD classifications are not complete and evidence-based. The need to define patients with a high risk of amputation and to categorize the patients whose prognosis improves owing to revascularization was indicated. Measurement of systolic toe pressure (STP), with a value below 30 mmHg suggesting the need for revascularization, appears to be an important factor. Patients with STP above 30 mmHg and suspected PAD should be treated conservatively in specialized centers, and the need for revascularization should be considered individually [16].

In physical examination, attention should be paid to changes in the color of the skin, especially to the paleness which increases after lifting the limb up. Long-lasting chronic ischemia is indicated by hair loss, especially in the case of unilateral ischemia as opposed to the healthy limb. Additional features include thickened, deformed, slow-growing nails with a tendency to fungal infections. Chronic ischemia of the lower limbs is also characterized by muscular atrophies affecting the muscles of the feet and shins. The Ratschow test is a useful adjunct to routine peripheral vascular assessment and, if positive, suggests more severe ischemia with distal limb artery involvement [17].

Location of the pain described by a patient may be useful for determining the anatomical area of the lesion, which may later facilitate diagnostic and therapeutic decisions. Claudication affecting the buttocks and thighs, sometimes combined with impotence, may be associated with stenosis or occlusion within the bifurcation of the abdominal aorta (Leriche's syndrome). The most frequent localization of PAD is the superficial femoral artery, and its narrowing is manifested by claudication within the calf. Exercise pain in the foot usually indicates impairment of the calf arteries—tibial anterior, tibial posterior, or sagittal (at least 2 of 3). Lesions may also occur in multiple sections of the arterial system, and it usually follows more advanced stage of atherosclerosis [18].

## 2. Conservative Treatment

Physiotherapists can start working with the patient with lower extremity PAD only if physical therapy is indicated by the angiologist or vascular surgeon after the patient has been diagnosed with lower extremity PAD; the location of the stenosis causing lower extremity ischemia has been detected; the stage of chronic ischemia, the initial and absolute claudication distances, and the type of treatment (pharmacological or surgical) are determined; and coexisting diseases and contraindications to specific rehabilitation methods are known. An individual personalized outpatient rehabilitation program should be designed for each patient.

### 2.1. Supervised Walking Training on the Treadmill

The current principles of atherosclerotic treatment of lower limb ischemia are based on the 2007 TASC II (Inter-Society Consensus for the Management of Peripheral Disease) guidelines and the 2017 ESC (European Society of Cardiology) guidelines, prepared in collaboration with the ESVS (European Society for Vascular Surgery) [19, 20]. Treatment of patients with intermittent claudication, especially with distal occlusion, is mainly conservative therapy, the aim of which is to improve the quality of life by relieving pain in the limbs while walking, thereby increasing the distance of claudication and reducing the risk of cardiovascular complications. Intermittent claudication is by far a greater indicator of cardiovascular risk than it is of the limb's fate. According to the literature, only 18% of patients with claudication will require surgical intervention and 10% will be amputated in a 10-year follow-up, while mortality from infarcts and strokes at 5, 10, and 15 years will be 30%, 50%, and 70%, respectively [21].

**Figure 1:**
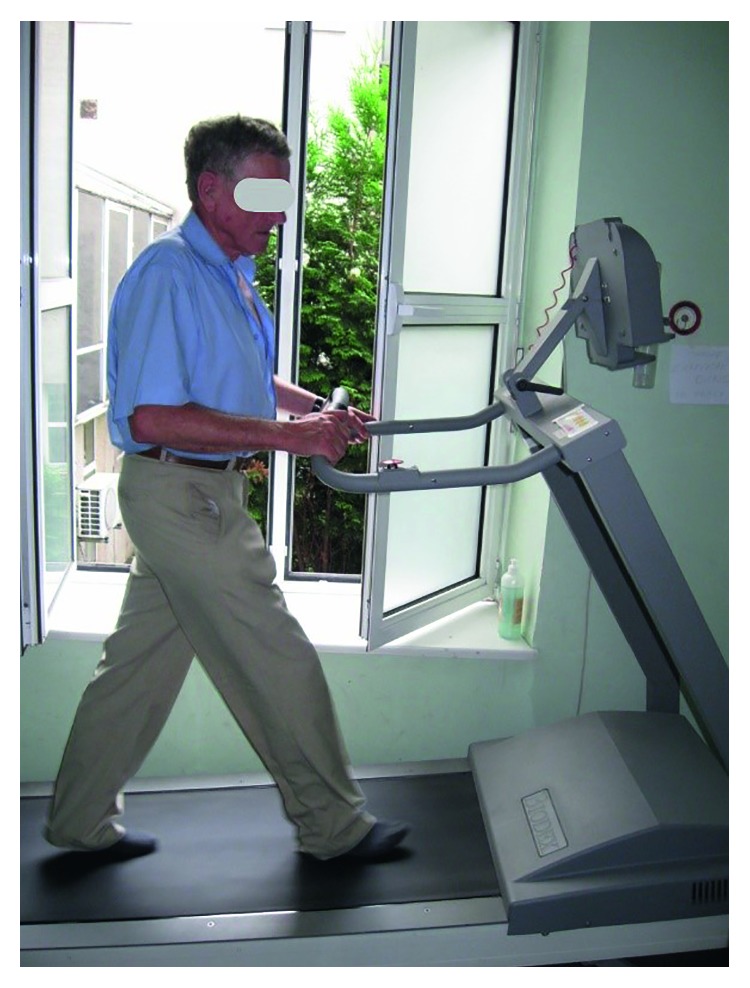
Treatment of claudication: treadmill training.

The walking training is a very important element in the conservative treatment of PAD, apart from pharmacotherapy and the modification of risk factors, which include smoking, lipid disorders, hypertension, diabetes, obesity, and stress. In accordance with the ESC and TASC II guidelines, the supervised training on a treadmill should be a primary procedure in all patients with intermittent claudication [22]. Training sessions should last 30 to 60 minutes and be run 3 times a week for a minimum of 3 months. The proposed belt speed is 3.2 km/h with increasing inclination of a treadmill. At the same time, it is emphasized that the maximum pain of ischemic muscles during walking should always be avoided [19, 20] (Figure 1). It remains a matter of dispute whether during the march any muscle pain should be allowed or not, considering the potentially unfavorable aspects of the ischemia—reperfusion phenomenon, which can lead to a generalized inflammatory reaction. The Gardner meta-analysis in 1995 focuses only on the benefits of increasing the distance of claudication, suggesting that walking training should be based on efforts conducted until maximum or close to the maximum severity of pain [23]. The current TASC II and the AHC (American Heart Association) and ESC guidelines suggest stopping the walking session when the pain reaches the moderate intensity, suggesting that if the patient stops at the onset of pain, the response to the training will be inadequate [24]. At the same time, TASC II strongly recommends avoiding the highest level of pain. Supervised walking training on a treadmill is the most effective form of rehabilitation of patients with intermittent claudication and is a “gold standard.” Moreover, it is a safe method because it practically excludes the risk of injuries or complications. The mechanism underlying the increasing walking distance in patients with intermittent claudication following exercise therapy is not clear. This effect is not based on one mechanism but results from a combination of many factors. Authors of many publications emphasize that treadmill walking causes beneficial rheological changes, causing increased deformability of erythrocytes and decreased blood viscosity. It also leads to morphological changes in muscle fibers, thanks to the improved capillary flow. Finally, training changes the perception of pain through increased supply of endorphins, leads to so-called “walking economy” improvement, and importantly causes pleiotropic changes in metabolism [25–27].

**Figure 2:**
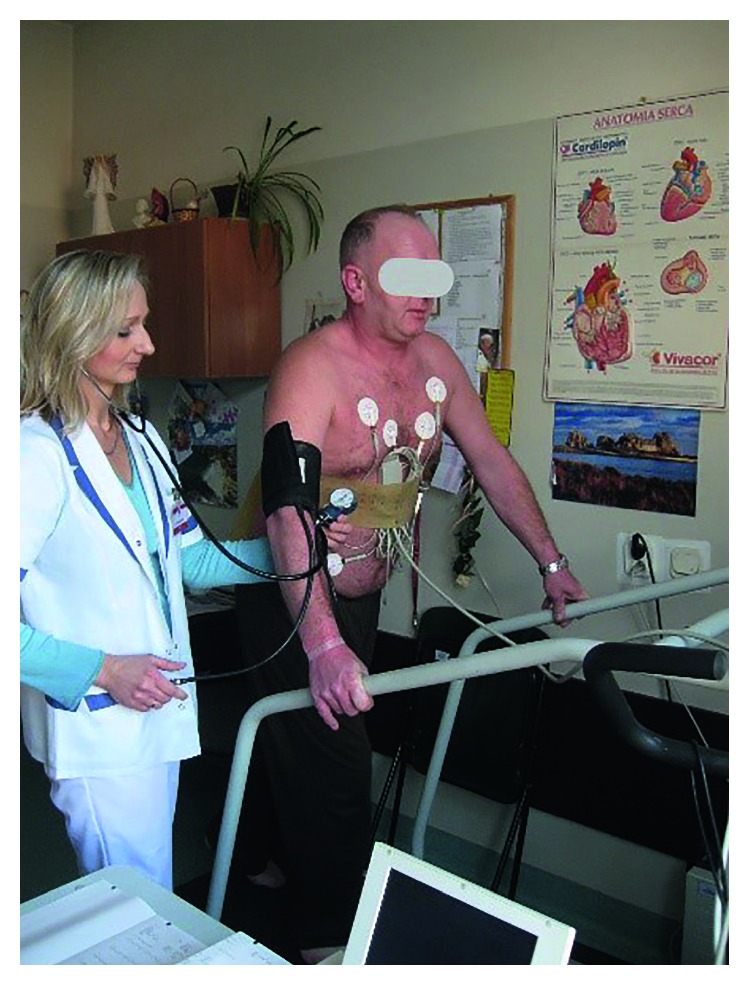
Exercise test on the treadmill with blood pressure (CTK), heart rate (CAS), and ECG monitoring.

Training on the treadmill can be gradually modified by extending the duration from 30 to 60 minutes as well as the speed from 3.2 to 4.8 km/h [28]. Atherosclerotic changes in arteries in patients with intermittent claudication are usually global; that is, they may also affect the cerebral and cardiac circulation. Therefore, treadmill exercises must take into account both beneficial (the extension of the claudication distance) and potential harmful effects on the cardiovascular system. The initial exercise test on the treadmill with measurements of blood pressure (CTK), heart rate (CAS), and ECG recording preceding long-term rehabilitation may allow to rule out severe concomitant circulatory disorders and thus avoid potential risk that a full, multiweek program of intense treadmill exercise might pose to affected individuals [29, 30] (Figure 2). Previously stated advanced unstable coronary heart disease, ischemic neurological disorders, and some muscular and joint disorders are also contraindications to motor rehabilitation on the treadmill. The percentage of patients disqualified from physical rehabilitation according to different tests varies from 7 to 34% [31, 32].

It should be noted that atherosclerosis is a systemic problem and cardiac rehabilitation is refunded by the national health services. At the same time, rehabilitation of patients with PAD in many countries is not eligible for refund.

### 2.2. Unattended Forms of Walking Exercises

Physical activity in patients with intermittent claudication is an integral part of general treatment. Since 69% of eligible PAD patients refuse to participate in supervised exercises 3 times a week because they are too burdensome for them, it is extremely important both to convince them about the purpose of walking exercises and to skillfully introduce training in their everyday activities [33]. In the literature, one can find papers discussing the principles of everyday activities of patients with claudication. It is assumed that the patient should walk about 3–5 km a day or ride a bicycle 10 km, stopping at the beginning of muscle pain, resting, and continuing training until the next stop [34, 35]. In practice, however, the vast majority of patients, instead of a detailed instruction on the walking training, receive at most a general recommendation “please do a lot of walking.” As the research shows, such a vague recommendation from a vascular surgeon or family physician, without explaining exactly what rules should be followed in everyday walks, can also lead to patients walking until maximum pain, which is inconsistent with the current TASC II recommendations [22]. Going to the maximum pain may precipitate adverse consequences and increases the risk of cardiovascular complications. Therefore, among patients with PAD, a safe “walk a lot, but not forcefully” recommendation should be promoted instead of using the wrong technique of “crying but walking” [36]. It should be emphasized that if during the walk the patient feels severe pain and muscle cramps, it causes highly adverse consequences as a result of ischemia—reperfusion injury (IRI) [37–39]. Restoration of the blood flow in severely ischemic muscles transfers active neutrophils to the general circulation with the subsequent release of active forms of oxygen and nitrogen (free radicals). These compounds cause, inter alia, the conversion of cholesterol into oxycholesterol, which is easily captured by the walls of the arteries through the scavenger receptor. This phenomenon to a certain extent can explain the rapid progression of atherosclerosis in patients with claudication and their high mortality due to myocardial infarction and stroke [8, 40].

On the contrary, it would also be a mistake to limit the patient's physical effort since the mere exchange of 10 minutes of a sedentary lifestyle for 10 minutes of low-intensity physical activity reduces mortality by as much as 9% [41]. The total exertion should consist of repeating marches, preferably until the beginning of lower extremity muscle pain, followed by rest. Maximum dilatation of arterial vessels occurs before ischemic pain. The advantage of walking training at home arises from the ability to individualize the effort; however, it is also easier for less disciplined patients to cease exercising without supervision. At the same time, it is emphasized that training at home can be as effective as outpatient treatment, provided that it is periodically monitored, for example, by activity-monitoring devices or by periodic checks every few days. Pain threshold should be determined in hospitals/clinics, which should be followed by discussion on activities that patient should repeat at home [42, 43]. Patients over 60 years are recommended to walk 60 steps per minute, whereas younger patients can go up to 120 steps per minute. A rhythmic and dynamic march is recommended. Slow walking is not recommended because part of the work is then used to stop, and consequently, it is nonergonomic activity. The gait should be smooth, and the tendency to save the affected limb (stalling) should be avoided. It is good when the march takes place on a moderately hard surface, preferably along a path in the park. If, of necessity, training is carried out on hard pavements, comfortable, airy footwear on a thick flexible sole is recommended. Duration of marching training should not exceed 20 minutes in the initial period, and later, it could be extended to 45 minutes. Training should be repeated 2-3 times a day. Patients with a longer claudication distance can be trained to climb stairs, which additionally involves crural and sciatic muscles [44]. Walking training can also be conducted in the form of Nordic Walking, where swinging symmetrical movements of the upper limbs are performed in a rhythmic walk with poles [45]. One of the recommended special forms of exercises is Buerger's training consisting in alternating exercises in the position of (1) ischemia, (2) congestion, and (3) relaxation of the muscles of the lower limbs [46].

### 2.3. Training on a Bicycle Ergometer

In the rehabilitation of patients with PAD, cycling and exercises on a bicycle ergometer (stationary bike) or a rehabilitation rotor, i.e., a device for exercising the lower limb muscles in a sitting position, are also used. These activities are particularly useful for patients who for some reason have limitations in conducting walking training, for example, degenerative joint changes in the lower limbs, neurological disorders, or excessive body weight. Because exercises on a bicycle ergometer with a comparable load increase the activity of the proximal muscle groups of the limb, while the march causes the load on the distal muscles—mainly calves, it is important that the patient places his forefoot, instead of the midfoot or the heel, on the pedal, which will allow for greater crural muscle involvement during exercise. If a patient is riding a bicycle or pressing the rotor pedals, it is not meant to make him or her breathless or evoke pain in the ischemic muscles of the lower limbs. Safe training on painless distances or until a small pain sensation interspersed with a few minutes of rest is recommended instead. The patient will benefit the most if the training is run every day and if the total distance covered about 10 km. The appropriately chosen load should be based on the result of an exercise test with CTK, CAS, and ECG recording, prior to the rehabilitation [24, 47].

### 2.4. Exercises of Upper Limbs

Training of upper arms (arm cranking) can be a highly beneficial procedure in patients with lower limb claudication according to some papers. It is suggested that improvement of cardiovascular endurance may contribute to improving gait. It has been demonstrated that exercises of upper limbs increase the antioxidant potential and lead to the extension of painless and maximum distance of claudication. Certainly, it can also be expected that this form of training contributes to strengthening the muscles of the upper limbs, possible blood pressure normalization, and improvements in the circulatory system as a whole [48–50]. The load should be chosen regarding optimal conditions for cardiovascular training. It is described as the physical fitness zone—defined as 50–75% of the maximum heart rate (220 *−* age). This range is considered to be the target heart rate during exercise. Exceeding this limit might pose a risk to the myocardium [51–53]. Patients with PAD who poorly tolerate walking or have a limited ability to perform lower limb exercises would benefit most from this form of activity.

### 2.5. Physical Medicine

Physical therapy is also used in the treatment of patients with intermittent claudication. It is not an isolated form of therapy but rather an accessory element of therapeutic regimen with primarily hyperemic, vasodilating, and analgesic and anti-inflammatory effects. This applies particularly to patients' concomitant conditions such as degenerative changes of the joints of the lower limbs, in which the ability of walking is deteriorated. These methods can also provide the patients with a preparatory element for muscle and joint kinesitherapy, as they result in tissue relaxation [54]. Previous studies are not conclusive as far as the efficiency of physiotherapeutic methods for increasing the distance of claudication or improving collateral circulation is concerned. Nonetheless, physiotherapy as adjunctive and complementary treatment in IIa/b limb ischemia in the Fontaine classification seems to be beneficial. It is worth noting, however, that because of the possible sensory disorders accompanying ischemia, the use of currents or thermal procedures should be carefully considered, so as not to cause burns [55].

The vasodilatation is achieved through the use of magnetotherapy, galvanic current, iontophoresis, Bernard's diadynamic currents, TENS, Träbert currents, and interference currents. The effect can also be obtained using infrared radiation, ultrasounds, and therapeutic baths, preferably carbon acid, radium, sulfide and hydrogen-sulfide, or brine [56].

The beneficial effect of tissue overheating on patients with obliterative atherosclerosis of the lower limb arteries is associated with an increase in blood flow. It can be achieved by means of infrared radiation of the Sollux lamp. These treatments also lead to relaxation of contractured muscles, joints, ligaments, and tendons.

In order to induce contraction of specific muscles, electrostimulation with electric impulses or Wadit high-frequency therapy is used for their stimulation. This type of electrogymnastics can be used in the treatment of muscular atrophy and muscular weakness, for example, in patients with short claudication distance [57].

The effect of biostimulation of microcirculation leading to restoration of the network of damaged blood vessels, improvement of the rheological properties of blood, and increased production of collagen by fibroblasts is observed during polarized light therapy. This speaks for the potential use of this physiotherapeutic method in the treatment of patients with intermittent claudication [58].

Analgesic and anti-inflammatory effects on muscles, joints, tendons, and nerves are obtained, thanks to the use of electrotherapy, including galvanic current and iontophoresis, diadynamic currents, interference currents, TENS, short-wave diathermy, and magnetotherapy. Light therapy with analgesic effects includes laser therapy, piler polarized light, and infrared radiation—Sollux lamp. Ultrasounds give a similar therapeutic effect [59].

The Kneipp method is used to reduce the activity of the vasomotor nerves resulting from regulation of the autonomic nervous system centers. This particular form of balneotherapeutic treatment in the case of atherosclerosis of the lower limbs is based on the comprehensive use of hydrotherapy (mainly Hauffe showers and baths), therapeutic use of physical activity, learning health-related lifestyle, phytotherapy, and mental hygiene. Exercises in water, in particular swimming, have a beneficial effect on the function of the heart and peripheral vessels. Faster circulation of blood contributes to better supply of tissues and organs with oxygen and nutrients and increases the exercise tolerance of the human body [60, 61].

## 3. Invasive Treatment

**Figure 3:**
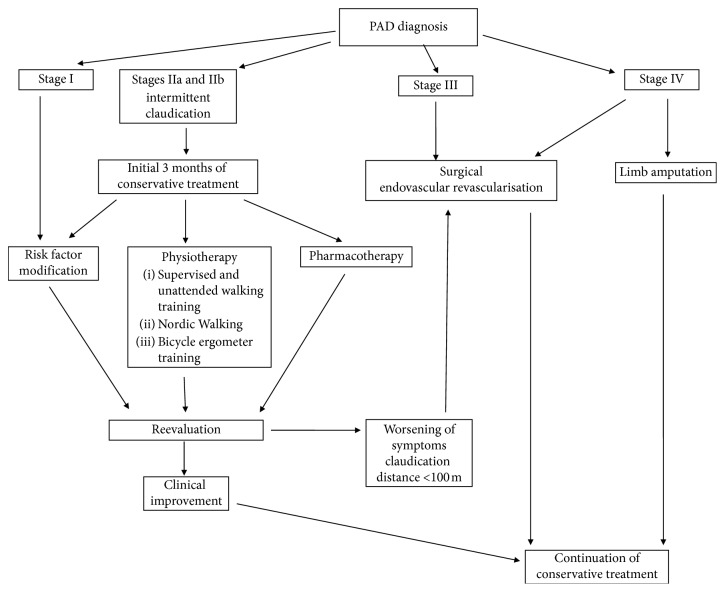
Management of PAD.

Endovascular or surgical invasive treatment is indicated only in a subgroup of patients with atherosclerotic lower limb ischemia, in whom motor rehabilitation and conservative treatment did not bring the expected improvement and in the subjects with critical limb ischemia due to the risk of amputation and even death. Generally, it is assumed that invasive treatment is undertaken when the distance of claudication is 100 m and below, as well as in the patients with resting pain and necrotic changes (III and IV degrees of Fontaine classification). The degree of impairment of the patient's mobility and quality of life, the anatomical location of atherosclerotic lesions, the expected immediate and remote benefits of treatment, and the risk of complications are the most important factors in the choice of therapeutic modality [62]. There are multiple scientific studies which show that a rigorous program of supervised exercise can be in the long term just as beneficial as angiosurgical treatment and is more effective than angioplasty [63] (Figure 3).

Percutaneous transluminal angioplasty (PTA) is an intravascular procedure and involves the widening of the narrowed or even occluded artery lumen. Very often, the stent implantation or the introduction of a specific “scaffolding” is an extension of balloon angioplasty. It is used to prolong the patency of the vessel after angioplasty and “hold” the atherosclerotic plaque or treat the dissection of the artery following ballooning.

Respiratory and antithrombotic exercises of lower legs, consisting in activation of the calf muscle pump, are a routine element of prophylaxis for hospitalized patients and constitute a valuable supplement to the rehabilitation of patients after endovascular procedures. Initially, the exercises involving the hip joint or the area of the puncture site (usually the femoral artery) are avoided so that the hematoma does not develop [64]. After the revascularization procedure, the inflow of blood to the limb improves significantly, resulting in limb warming, increase in the ankle-arm index, elongation of painless and maximal walking distance, reduction of rest pain, and quality of life improvement. Endovascular procedures must be followed with auxiliary measures, that is, intake of antiplatelet or antiaggregating drugs and cholesterol-normalizing drugs and normalization of blood pressure and blood glucose levels [20]. Not only systematic pharmacotherapy but also adequate physical activity and nicotine abstinence are necessary to maintain long-term treatment results. Procoagulative effects of smoking significantly increase the risk of reocclusion of the treated artery, and loss of the limb may follow. Physical activity recommendations after endovascular surgery and hospital discharge are akin to these for the patients with intermittent claudication and mainly involve walking training 3–5 km per day consisting of several stages followed by rest when low-intensity pain occurs [65]. A 6-minute test or a hall-walk test is the simplest measure for the assessment of the claudication distance (both PFWD and MCD) increase after arterial repair and rehabilitation. It is believed that the exertion during this examination is more representative of daily activities compared to other forms of distance assessment, e.g., a treadmill test [66, 67].

## 4. Therapeutic Education

Proper education of patients with atherosclerosis of the lower limbs should be a professional and individualized action and cannot be based solely on spreading of the old Housley's principle “stop smoking and keep walking” [68].The patient's conscious participation in the therapeutic process increases the effectiveness of treatment. The PAD patient should know that correctly conducted, long-term training is an indispensable element of therapy and that smoking is of great importance in the development of atherosclerosis [69]. Nicotine stimulates the adrenal medulla causing the secretion of catecholamines which results in, among others, vasoconstriction, increase of peripheral vascular resistance, and reduction of HDL (“good”) cholesterol. Depending on the filter, cigarettes may vary as far as amounts of nicotine and tar components are concerned, but still, the carbon monoxide generated by tobacco combustion has the most significant damaging effects on the vascular endothelium. Its amount generated during smoking is always the same, regardless of cigarettes being “light” or “strong.” The risk of atherosclerosis increases with the amount of cigarettes smoked and the time of smoking. The mere cessation of smoking may result in an increase in the distance of claudication by as much as 40% [70]. The introduction of a proper diet is an important activity that should be carried out by patients with PAD. The most important purpose of nutritional regimen is to reduce cholesterol, especially LDL cholesterol. Limitation of the intake of saturated fatty acids contained especially in milk and meat products is one of the preventive measures for lowering the level of LDL and triglycerides and increasing HDL. At the same time, the consumption of vegetable oils rich in unsaturated fatty acids is recommended. Rapeseed oil, omega-6 fatty acid-rich sunflower or soybean oil, and omega-3-abundant linseed and walnut oil are the examples [6]. Another preventive measure is the supplementation of nitric oxide. It can be achieved by the intake of nitrate-rich products, which then undergo reduction to nitric oxide *in vivo*. A popular product rich in nitrates is beet juice. It can be assumed that the combination of beet juice intake and walking training would bring the greatest benefit to patients with PAD [71].

Diabetes is an important risk factor for the development and progression of PAD. Among diabetic patients, intermittent claudication occurs 4 times more frequently, whereas gangrene and limb amputation as a consequence peripheral atherosclerosis occur 15 times more often than in nondiabetic subjects [64, 72]. Therefore, in these patients, besides maintaining blood glucose within the recommended limits and the use of individually selected physical exercises, education is necessary. It includes regular feet inspection and hygiene, proper toenail cutting, wearing appropriate footwear, insoles and socks, and avoiding mechanical and thermal injuries [73].

## 5. Conclusions

PAD is a systemic disease. Symptomatic PAD not only poses the risk to ischemic extremity, but above all, it is an important prognostic factor of general cardiovascular complications. Rehabilitation of patients with intermittent claudication should be a comprehensive, long-term action, initiated from the moment of diagnosis. It should be individually matched to the patient's capability. A trained and motivated patient has a chance to improve both the life quality and expectancy, which is why education of patients with PAD is extremely important. Effective education should be conducted not only by angiologists or vascular surgeons but also by physiotherapists and nurses.
